# *Angiostrongylus cantonensis* infection in molluscs in the municipality of São Gonçalo, a metropolitan area of Rio de Janeiro, Brazil: role of the invasive species *Achatina fulica* in parasite transmission dynamics

**DOI:** 10.1590/0074-02760150106

**Published:** 2015-09

**Authors:** Ana PM Oliveira, Rosana Gentile, Arnaldo Maldonado, Eduardo J Lopes Torres, Silvana C Thiengo

**Affiliations:** 1 Fundação Oswaldo Cruz, Instituto Oswaldo Cruz, Programa de Pós-Graduação em Biologia Parasitária, Rio de Janeiro, RJ, Brasil; 2 Fundação Oswaldo Cruz, Instituto Oswaldo Cruz, Laboratório de Biologia e Parasitologia de Mamíferos Silvestres Reservatórios, Rio de Janeiro, RJ, Brasil; 3 Universidade do Estado do Rio de Janeiro, Faculdade de Ciências Médicas, Departamento de Microbiologia, Imunologia e Parasitologia, Laboratório de Helmintologia Romero Lascasas Porto, Rio de Janeiro, RJ, Brasil; 4 Fundação Oswaldo Cruz, Instituto Oswaldo Cruz, Laboratório de Malacologia, Rio de Janeiro, RJ, Brasil

**Keywords:** cerebral angiostrongyliasis, Bradybaena similaris, helminths’ larvae, intermediate hosts

## Abstract

The aim of this study was to analyse the infection dynamics of*Angiostrongylus cantonensis *in its possible intermediate hosts over two years in an urban area in the state of Rio de Janeiro where the presence of*A. cantonensis* had been previously recorded in molluscs. Four of the seven mollusc species found in the study were exotic.*Bradybaena similaris *was the most abundant, followed by*Achatina fulica*, *Streptaxis *sp.,* Subulina octona*, *Bulimulus tenuissimus*, *Sarasinula linguaeformis *and* Leptinaria unilamellata*. Only* A. fulica *and* B. similaris *were parasitised by* A. cantonensis* and both presented co-infection with other helminths. The prevalence of *A. cantonensis*in* A. fulica *was more than 50% throughout the study. There was an inverse correlation between the population size of*A. fulica *and the prevalence of* A. cantonensis *and abundance of the latter was negatively related to rainfall. The overall prevalence of* A. cantonensis *in *B. similaris*was 24.6%. *A. fulica *was the most important intermediary host of* A. cantonensis *in the studied area and*B. similaris *was secondary in importance for* A. cantonensis *transmission dynamics.

*Angiostrongylus cantonensis* (Chen, 1935), the rat lungworm, is a parasitic nematode discovered in the pulmonary arteries and hearts of domestic rats in China (Wang et al. 2008). It cans parasitise the central nervous system of humans causing eosinophilic meningitis. The disease is known as cerebral angiostrongyliasis (Hsu et al. 1990,Ismail & Arsura 1993) or, commonly, rat lungworm disease (RLD). The first record of angiostrongyliasis caused by *A. cantonensis* infection was reported in 1945, by Nomura and Lin, based on the observation of a nematode in the cerebrospinal fluid of a patient (Prociv & Carlisle 2000). Since then, RLD, which is endemic in Southeast Asia and the Pacific islands, has been reported in more than 30 countries worldwide, especially in tropical and subtropical regions (Kim et al. 2014). Among 2,800 cases of RLD, 77% occurred in Southeast Asia, China and Japan (Wang et al. 2008). In Brazil, RLD is an emerging disease first reported in 2006 and infected hosts were observed in the states of Rio de Janeiro (RJ), Espírito Santo (ES), Pernambuco (PE), São Paulo, Rio Grande do Sul and Paraná (Caldeira et al. 2007, Lima et al. 2009, Espírito-Santo et al. 2013,Morassutti et al. 2014).

The life cycle of *A. cantonensis* is complex and involves rats as definitive hosts, molluscs as intermediate hosts and crustaceans, frogs, fishes and other invertebrates as paratenic hosts. Several gastropod species from various families have been reported as intermediate hosts of *A. cantonensis* (Kim et al. 2014), including *Achatina fulica*, Browdich, 1822, popularly known as the African giant snail (Mead 1961, Alicata 1966, Tsai et al. 2013, Morassuti et al. 2014). *A. fulica*was included on a list of 100 of the worst invasive species in the world (Lowe et al. 2004). In Brazil it was introduced for commercial purposes in the 1980s and currently is found in 25 of the 26 Brazilian states (Thiengo et al. 2007), usually forming dense populations. *A. fulica* and other molluscs naturally infected with*A. cantonensis* have been described in various regions of Brazil byThiengo et al. (2010), Maldonado Jr et al. (2010), Carvalho et al. (2012) and Moreira et al. (2013).

In Brazil, the emergence of RLD cases associated with the introduction and spread of*A. fulica* requires a better understanding of RLD transmission dynamics. The aim of this study was to analyse infection dynamics of *A. cantonensis* in its possible intermediate hosts in an urban area in RJ where the presence of *A. cantonensis* in molluscs has been recorded.

## MATERIALS AND METHODS

**Fig. 1:**
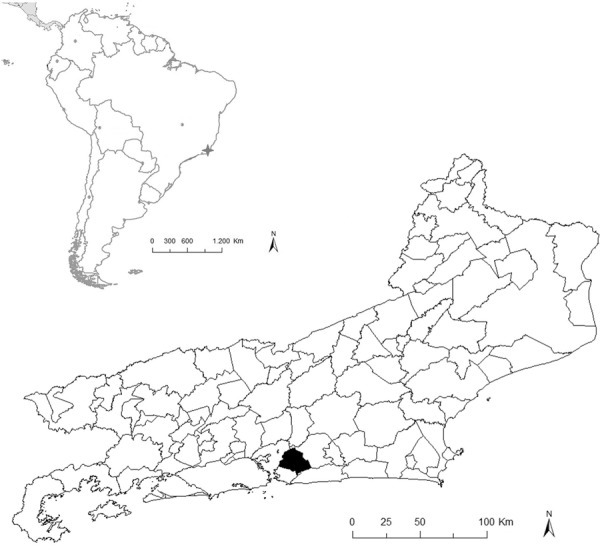
: location of the state of Rio de Janeiro, Brazil, and the municipality of São Gonçalo (black) within the state.

*Study area* - This study was carried out in Trindade locality (22º49’37”S 43º03’14”W), which is a district of the municipality of São Gonçalo (Fig. 1), the second most populous city in RJ with approximately one million inhabitants (IBGE 2010). The climate is warm humid tropical with hot humid summers and mild dry winters. The annual average temperature is 25ºC. Maximum average temperature during the study period was 30.9ºC in February 2010 and the minimum average temperature was 21ºC in June 2010 and July 2011. The annual average rainfall is 120 mm and varied during the study period from 13.9 mm in September 2011 to 341.2 mm in March 2010 (data obtained from São Gonçalo Urban Climatological Station from Geosciences Laboratory, Rio de Janeiro State University).

The study site comprised an area of 75 m^2^ divided into three plots of 5 x 5 m in grassy vegetation with a predominance of razor grass and a few bushes in the backyards of human dwellings where the presence of *A. cantonensis*had been recorded (Maldonado Jr et al. 2010). The plots were bordered by a pavement way, a dwelling wall, a small stream and a very narrow strip of grass vegetation continuing along the backyards of the dwellings. In two plots, vegetation covered the ground completely and the third plot had open areas with exposured clay soil.

*Field and laboratory methods* - Molluscs were sampled on 11 occasions in all seasons from January 2010-October 2011. On each occasion, specimens were collected manually on one day, always in the morning. Specimens were taken to the laboratory and kept in a terrarium (20 x 19 x 30 cm) with 3 cm of autoclaved moist clay on the bottom, under controlled temperature (23 ± 2ºC) and fed fresh lettuce leaves every other day. Mollusc identification followed Boffi (1979), Araújo et al. (1960) and Thomé and Gomes (2006).

Larvae of helminths were recovered from the molluscs using a 0.7% HCl artificial digestion method (Graeff-Teixeira & Morera 1995). Larvae were fixed in ethanol alcohol 70%, formaldehyde 40% and acetic acid ≥ 99.85% glacial and identified using a light microscope according toAnderson et al. (2009) and Ash (1970). Larvae recovered from each mollusc specimen were counted only for *A. fulica*. To confirm identification of *A. cantonensis* specimens, recovered worms were administered orally to rats (*Rattus norvergicus*) in captivity. After 35 days, adult worms were recovered from the pulmonary arteries of the rats and identified by morphometry according to Maldonado Jr et al. (2010) and by molecular techniques according to Monte et al. (2012). This procedure was carried out following the Ethical Committee on Animal Use of Oswaldo Cruz Foundation (license 100/2011).

*Data analysis *- Mollusc abundance was defined as the number of specimens of each species collected. Mollusc species richness was defined as the overall number of species sampled.

The prevalence of each helminth species was calculated for each month for all mollusc species and prevalence was defined as the proportion of infected molluscs for a given helminth species in relation to the total number of molluscs analysed for a given mollusc species, according to Bush et al. (2001). To investigate any possible immediate relationship between climatic variables and helminth prevalence, the influence of climatic variables on prevalence of *A. cantonensis* in *A. fulica* and in*Bradybaena similaris *(the molluscs species in which *A. cantonensis* was found) was investigated using multiple linear regressions, with a one-month time lag. To investigate if mollusc abundance and*A. cantonensis* prevalence varied in similar or opposing ways, simple correlations were carried out between these parameters.

Abundance and intensity of *A. cantonensis* in *A. fulica* were calculated for each month according to Bush et al. (2001). Abundance was defined as the number of helminths recovered divided by the total number of molluscs analysed. Intensity was defined as the number of helminths recovered divided by the number of infected molluscs. The dispersion index for *A. cantonensis* in*A. fulica* was calculated as the variance to mean ratio of parasite abundance. We used simple correlations to investigate whether *A. fulica* abundance and *A. cantonensis* abundance and intensity varied in similar ways through time. To verify possible influence of*A. fulica* shell size on abundance and prevalence of *A. cantonensis* we used simple linear regressions. Shell size was defined as length of the mollusc from the apex to the shell opening.

Data on mollusc abundance, parasitological parameters and climatic variables were tested for normal distribution using the Shapiro-Wilk test. Significance level in all analyses was considered as α < 0.05. Tests were performed using PASW Statistics v.18 and PAST v.2.10.

## RESULTS

*Mollusc community *- The mollusc community was composed of seven species (overall species richness) and it did not vary among plots. Monthly species richness varied from one-six, with highest values during the winter months. In total, 467 individuals were collected. *B. similaris* was the most abundant species, with 245 individuals, representing 52.4% of all molluscs collected. *A. fulica* was the second most abundant (153 individuals, 32.7%). Other species occasionally present were *Streptaxis* sp. (22 individuals, 4.7%), *Subulina octona* (11 individuals, 2.3%),*Bulimulus tenuissimus* (d’Orbigny, 1835) (9 individuals, 1.9%),*Sarasinula linguaeformis* (3 individuals, 0.6%) and*Leptinaria unilamellata* (d’Orbigny, 1835) (2 individuals, 0.4%).

**Fig. 2:**
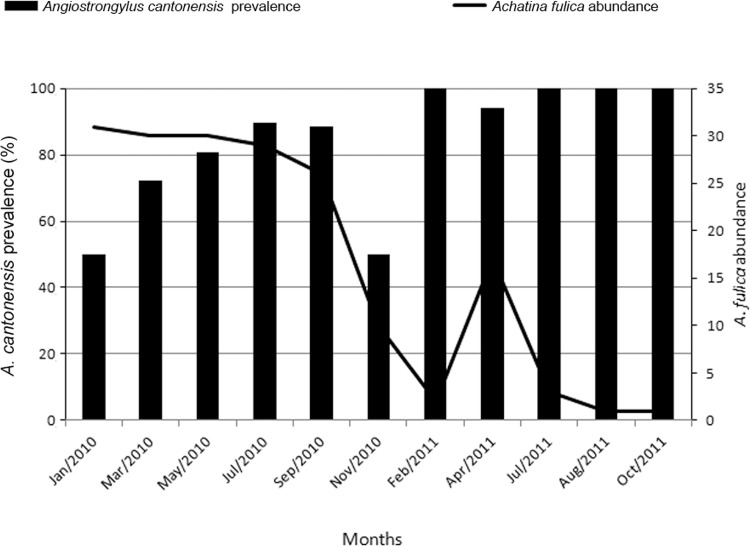
: *A. fulica* abundance (right axis) and *A. cantonensis* prevalence in this host (left axis) through time in Trindade, municipality of São Gonçalo, state of Rio de Janeiro, Brazil.

**Fig. 3:**
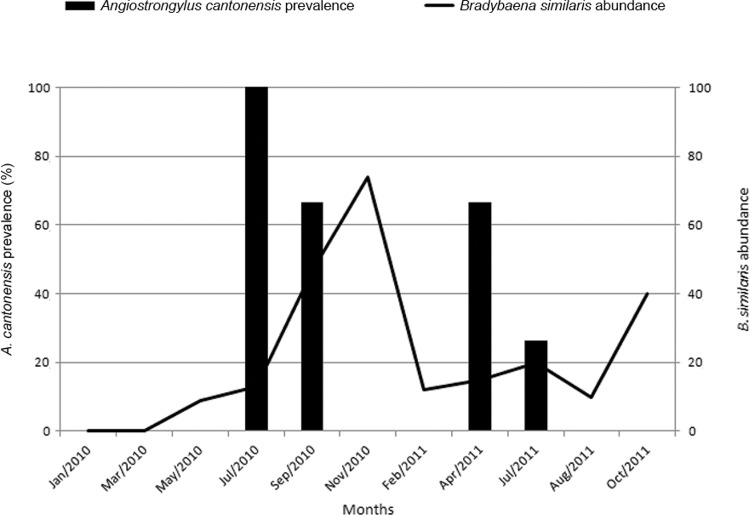
: *B. similaris* abundance (right axis) and *A. cantonensis* prevalence in this host (left axis) through time in Trindade, municipality of São Gonçalo, state of Rio de Janeiro, Brazil.

*A. fulica* was the only species present in all samples. Its abundance decreased during the study period, except for in April 2011, and it did not exhibit a clear annual pattern of abundance (Fig. 2).*B. similaris* abundance was highest in September and November 2010 (Fig. 3). There was a negative correlation between *A. fulica* and *B. tenu- issimus*abundances (*rs* = -0.619, p = 0.042, n = 11).

*Helminth parasitism on molluscs* - Helminth presence was assessed in 270 mollusc specimens. Three nematode species were found: *A. cantonensis*, *Rhabditis* sp. and*Strongyluris* sp. *A. cantonensis* had an overall presence of 78.7% in *A. fulica* and an overall prevalence of 24.6% in *B. similaris*. *Rhabditis* sp. was present in all mollusc species, except *L. unilamellata,* and had an overall prevalence of 14.2% in *A. fulica,* 68.6% in *B. si- milaris,*20% in *S. octona*, 31.8% in *B. tenuissimus*, 33.3% in *Sarasinula *sp. and 9.5% in*Streptaxis *sp. *Strongyluris* sp. occurred only in *A. fulica *and in *B. similaris* with overall prevalence of 13.5% and 2.12%, respectively.

*A. cantonensis infection in molluscs - *Prevalence of *A. cantonensis* on *A. fulica* was always more than 50% (Fig. 2) and showed a negative correlation with *A. fulica* abundance (*rs* = -0.801, p = 0.003, n = 11). Monthly mean abundance of *A. cantonensis* in *A. fulica* was 124.3 larvae per host varying from 14 in February 2011 to 416.8 in January 2010. Monthly mean intensity of *A. cantonensis* in*A. fulica* was of 220.5 larvae per infected host, varying from 14 in February 2011 to 833.6 in January 2010. *A. cantonensis*abundance and intensity were not correlated with *A. fulica*abundance (*rs* = 0.201, p = 0.535, n = 11; *rs* = 0.420, p = 0.198, n = 11, respectively). However, *A. cantonensis*abundance was related negatively with rainfall (*R* = 0.788, b = -0.951, p = 0.007). Prevalence and intensity were not related with any climatic variable. Distribution of *A. cantonensis* was highly aggregated among *A. fulica* individuals with a dispersion index of 952.81 and the highest absolute abundance in one mollusc was 3,108 larvae.

Overall prevalence of *A. cantonensis* in *B. similaris* was 24.6%, varying from 0-100%. This helminth was found in*B. similaris* only in July 2010, September 2010, April 2011 and July 2011 (Fig. 3). There was no relation between prevalence and any climatic variable (*R* = 0.369, p = 0.294). There was no correlation between *A. cantonensis* prevalence and *B. similaris* abundance (*r* = 0.035, p = 0.918, n = 11) nor between *A. cantonensis* prevalence in *A. fulica* and *A. cantonensis* prevalence in *B. similaris* (*r* = 0.276, p = 0.412, n = 11). Abundance and intensity in *B. similaris* were not calculated because the snails were pooled for digestion and larvae were not counted.

Shell size of the *A. fulica* specimens collected varied from 1-9 cm, with a mean of 4.35 cm and a standard deviation of 1.67. There was no significant correlation between *A. fulica* shell size and helminth abundance (*R* = 0.029, p = 0.727, n = 142), nor between monthly median shell size and *A. cantonensis* monthly prevalence (*R* = 0.499, p = 0.118, n = 10).

## DISCUSSION

Four of the seven mollusc species found in the study are considered exotic (*A. fulica*, *B. similaris*, *L. unilamellata* and *S. octona*) and three are native (*B. tenuissimus*, *S. linguaeformis* and*Streptaxis *sp.) (Simone 2006). These species are widely distributed and some play a role in transmission of helminth parasites of medical and veterinary importance (Souza & Lima 1990). The invasive species*A. fulica* stands out for its recent introduction and for rapidly spreading throughout Brazil (Thiengo et al. 2007, Zanol et al. 2010), including RJ (Carvalho et al. 2012). In the present study, abundance of *A. fulica* and *B. similaris* were higher than in other studies conducted in *A. cantonensis* transmission areas in Brazil. Carvalho et al. (2012) studied mollusc fauna in nine Brazilian ports, finding *S. octona* to be most abundant, followed by*A. fulica* and *B. similaris*. The same was found by Caldeira et al. (2007) in ES. Thiengo et al. (2010) reported a predominance of *L. unilamellata*, *Sarasinula marginata* and*A. fulica* in PE and did not find *B. similaris*.

*A. fulica* and *B. similaris*, originating in Africa and Asia, respectively, have been introduced to many regions of the world by human activities. In Brazil, they occur in nearly all states (Oliveira et al. 1990, Zanol et al. 2010). They spread easily to various environments and can cause major health and economic effects, either as agricultural pests or as intermediate hosts of helminths. In addition, they are able to establish readily in urban and periurban areas, invading gardens, vegetable gardens and wastelands. These characteristics may have resulted in higher abundance of *A. fulica* and *B. similaris* compared to other mollusc species in the area studied. Exotic invasive species tend to dominate the natural communities where they establish (Simone 2006).

We observed a marked increase in *B. similaris* abundance in spring and summer. These results are according to Araújo (1989), who found seasonal occurrence of this species with activities after periods of heavy rainfall, restricting its appearance to relatively short periods of the year. Leahy (1980, 1983) found high resistance to environmental changes by *B. similaris* and ability to survive to desiccation for up to 24 days and to return to normal activity when replaced in moist environments with food. In general, terrestrial pulmonate molluscs tend to be more active in the rainy season, when relative humidity of air and soil are higher (Pérez et al. 2008). Mollusc species richness did not vary among plots, but varied over time, increasing in the winter months, especially due to the accidental species *Streptaxis *sp.*, L unilamellata* and *S. linguaeformis* moving from adjacent vegetation, which seemed to be dependent on seasonal variations.

The present study is the first report of the occurrence of *A. cantonensis* in *B. similaris* in RJ, although *B. similaris* has been identified as an intermediate host of this nematode elsewhere (Caldeira et al. 2007, Carvalho et al. 2012). Results of the present study indicate *A. fulica* and *B. similaris* as the most important intermediate hosts of *A. cantonensis* in Trindade, with much higher prevalence in *A. fulica* than in *B. similaris*. In addition, results indicate an important role of*A. fulica* in the transmission cycle of *A. cantonensis*, because this species was found infected throughout the year at high prevalence rates. *A. fulica *seems to be susceptible to various parasite species, which corroborates its importance as a helminth intermediate host. Furthermore, its body size and high abundance may favour parasite occurrence. Larger hosts may offer more space and diversity of microhabitats and they are able to support a greater richness of parasite species.

Prevalence and abundance of *A. cantonensis* increased most during and at the end of the dry season. In November 2010, as *A. fulica*abundance decreased, the prevalence of *A. cantonensis* dropped. At this point, *B. similaris* abundance was highest and in both 2010 and 2011, the highest prevalence of *A. cantonensis* on this host species occurred during the first months of *A. fulica* abundance decrease. Moreover, even with lower abundance of *A. fulica* in 2011, prevalence of the nematode in this host always was high. These observations suggest*B. similaris* as the second most significant host for the*A. cantonensis* transmission cycle. Although this mollusc was the most abundant in the present study, *A. cantonensis *infection was observed only during dry months. Carvalho et al. (2012) showed that, in Brazilian port areas, the rate of infection of*A. cantonensis* in *B. similaris* was 100%, in*S. marginata*, 84%, in* S. octona*, 76% and in*A. fulica*, 66%. They concluded that *B. similaris* had importance equal to or greater than *A. fulica* in the parasite transmission cycle. In the present study, it is possible that *B. similaris* acted as an auxiliary intermediate host for the parasite transmission. It is important to point out that the present study was carried out over nearly two years, while Carvalho et al. study (2012) was less protracted.

The dispersion of parasites is of great importance to understanding parasite-host dynamics (De Jong 1976, Dobson & Roberts 1994). We observed a highly aggregated distribution pattern of *A. cantonensis* in *A. fulica* population, indicating that unequal exposure of hosts and differences in individual susceptibility to infection may have influenced this pattern and resulted in many hosts harbouring few or no parasites and a few hosts harbouring the bulk of parasites as expected for most parasite populations (Poulin 2007).

Although the highest mean parasite load of *A. cantonensis* in*A. fulica* was observed in the older age group, we did not observe a significant relationship between *A. fulica* shell size and abundance of *A. cantonensis, *suggesting that helminth larvae infection did not depend on mollusc shell size. Sithithaworn et al. (1991) demonstrated that *A. cantonensis* prevalence may increase with *A. fulica* age and hosts of up to 200 days reached a 50-60% infection level, with total prevalence of 53% at this age and size. Thus, in the present study, the microenvironment may have influenced infection more than shell size, due to high degree of spatial aggregation of parasites in the *A. fulica* population.

Recent studies have reported *A. cantonensis* infection in Brazil in*Rattus rattus* and *R. norvergicus,* with both acting as definitive hosts (Simões et al. 2011, Moreira et al. 2013). In the studied area, infected *R. norvergicus* were observed to have high prevalence and abundance of *A. cantonensis* and to contribute to dispersing the parasite to new areas (Simões et al. 2014).

In the present study, *A. fulica* played an important role as intermediate host in the *A. cantonensis* transmission dynamics, due to its high abundance and high infection rates, regardless of season. *B. similaris* was a second most important to *A. cantonensis* transmission dynamics, mostly when population abundance of*A. fulica* was low. The high abundance of these molluscs observed associated with high prevalence of *A. cantonensis *and the presence of infected rodents may enable transmission of this nematode in the locality throughout the year.
